# Comprehensive Transcriptome Analysis Reveals Competing Endogenous RNA Networks During Avian Leukosis Virus, Subgroup J-Induced Tumorigenesis in Chickens

**DOI:** 10.3389/fphys.2018.00996

**Published:** 2018-07-26

**Authors:** Lingling Qiu, Guobin Chang, Zhiteng Li, Yulin Bi, Xiangping Liu, Guohong Chen

**Affiliations:** ^1^Key Laboratory of Animal Genetics and Breeding, Molecular Design of Jiangsu Province, Yangzhou University, Yangzhou, China; ^2^Poultry Institute, Chinese Academy of Agricultural Sciences, Yangzhou, China

**Keywords:** subgroup J avian leukosis virus, tumorigenesis, competing endogenous RNA, lncRNA, network

## Abstract

Avian leukosis virus subgroup J (ALV-J) is an avian oncogenic retrovirus that induces myeloid tumors and hemangiomas in chickens and causes severe economic losses with commercial layer chickens and meat-type chickens. High-throughput sequencing followed by quantitative real-time polymerase chain reaction and bioinformatics analyses were performed to advance the understanding of regulatory networks associated with differentially expressed non-coding RNAs and mRNAs that facilitate ALV-J infection. We examined the expression of mRNAs, long non-coding RNAs (lncRNAs), and miRNAs in the spleens of 20-week-old chickens infected with ALV-J and uninfected chickens. We found that 1723 mRNAs, 7,883 lncRNAs and 13 miRNAs in the spleen were differentially expressed between the uninfected and infected groups (*P* < 0.05). Transcriptome analysis showed that, compared to mRNA, chicken lncRNAs shared relatively fewer exon numbers and shorter transcripts. Through competing endogenous RNA and co-expression network analyses, we identified several tumor-associated or immune-related genes and lncRNAs. Along transcripts whose expression levels significantly decreased in both ALV-J infected spleen and tumor tissues, *BCL11B* showed the greatest change. These results suggest that *BCL11B* may be mechanistically involved in tumorigenesis in chicken and neoplastic diseases, may be related to immune response, and potentially be novel biomarker for ALV-J infection. Our results provide new insight into the pathology of ALV-J infection and high-quality transcriptome resource for in-depth study of epigenetic influences on disease resistance and immune system.

## Introduction

Avian leukosis virus (ALV) is a highly oncogenic retrovirus and can be classified as endogenous virus (subgroup E) or exogenous virus (subgroups A, B, C, D, and J), on the basis of viral envelope interference, host range, mode of transmission, and cross-neutralization patterns (Payne et al., 1991). ALV-J virus was first obtained from meat-type chickens in 1988 (Payne et al., 1993). ALV-J infection can induce tumors and enhance the susceptibility to secondary infection and result in enormous financial ruin in poultry industry around the world (Payne and Nair, 2012). Recently, ALV-J infection has become epidemic in China and has induced severe outbreaks in both commercial layer chickens and meat-type chickens (Cui et al., 2006; Gao et al., 2012; Liu et al., 2016). ALV-J-infected layer chickens commonly appear significant egg yield declines and hemorrhaging in the skin around the phalanges and feather follicles. The morbidity and mortality rates caused by ALV-J infection have reached 60 and 20%, respectively, in some flocks in China (Gao et al., 2010).

Long non-coding RNAs are non-coding transcripts with size larger than 200 nucleotides (Mattick, 2001). LncRNAs were once viewed as transcriptional “noise” without biological functions (Ponting and Belgard, 2010), but emerging evidence suggests that they function as key regulators in plentiful biological processes, for example, genomic imprinting, cell differentiation, cycle progression, apoptosis, and immune responses, as well as different types of cancer (Ran et al., 2016; Schein et al., 2016; Chen et al., 2017; Zhou et al., 2017). Accumulating evidence indicates that lncRNAs may play roles in regulating oncogenes or suppressors in human cancer (Yochum et al., 2007; Liu et al., 2013; Xu et al., 2014), and as regulators in physiological and pathological responses (Geisler and Coller, 2013; Maass et al., 2014).

In the immune system, lncRNAs function in innate immune (Carpenter et al., 2013; Rapicavoli et al., 2013), in adaptive immunity (Liu et al., 1997; Willingham et al., 2005), and in host defenses against microbial infection (Vigneau et al., 2003; Gomez et al., 2013). The knowledge of the molecular functions of lncRNAs is expanding rapidly. Several previous studies provide convincing evidence that lncRNAs participate in regulating gene expression by targeting either splicing, decay, or translation of target mRNAs (Rinn and Chang, 2012; Ulitsky and Bartel, 2013) or by binding miRNAs and reducing its availability to bind mRNA targets to serve as ceRNAs (Salmena et al., 2011; Tay et al., 2014). However, whether the ceRNA network is also involved in the tumorigenesis induced by ALV-J infection in chickens remains unclear.

In this study, we constructed mRNA-associated ceRNA networks and identified several tumor-associated or immune-related genes by analyzing three sequence data sets in ALV-J-infected chicken spleen samples. We then validated a subset of this network by qRT-PCR, in which the greatest changes occurring between ALV-J-infected liver tumor and adjacent normal tissues, and a ceRNA network mediated by *BCL11B* get our attention. These findings illuminate a novel mechanism and biomarker for ALV-J-induced tumorigenesis in chickens.

## Materials and Methods

### Ethics Statement

All experimental procedures were performed in accordance with the Regulations on The Administration of Experimental Animals issued by the Ministry of Science and Technology in 1988 (last modified in 2001, Beijing, China). All experimental animal operations were approved and guided by the Animal Care and Use Committee of Yangzhou University.

### Sampling, Pathological, and Genetic Review

Twenty-week-old female black-bone silky fowls (BSFs) with or without spontaneous ALV-J infection, were obtained from the progenitor breeding chicken farm of Lihua Animal Husbandry, Co. Ltd. (Jiangsu, China). The clinical symptoms of ALV-J infection were reviewed as depression, hemorrhages in the phalanges skin, splenomegaly and hepatomegaly with tumor nodules by independent pathologists. The spleens of infected BSFs were enlarged by up to several times the normal size. Spleen samples of uninfected chickens and chickens suspected of having ALV-J infection were collected independently, washed in RNase-free water, transferred to tissue-cryopreservation tubes, immediately submerged in liquid nitrogen, and stored until genetic analysis was performed.

The polymerase chain reaction (PCR) was performed to test genomic DNA extracted from spleen samples. Specific primers were designed according to previously studies, synthesized by Shanghai Sangon Biotechnology Company (Shanghai, China), and used to detect endogenous and exogenous ALVs (Smith et al., 1998) and other tumorigenic virus in avian: MDV (Ding et al., 2007) and REV (Ji et al., 2001). The sequences of all sets of primers are showed in **Table 1**. Finally, the spleens were divided into two groups (ALV-J-positive group: ALV-J^+^; ALV-J-negative group: ALV-J^-^) and subjected to RNA-Seq and small RNA-Seq. In the meantime, we also chose additional chickens for pathological and genetic review and then collected tumor tissues and adjacent normal tissues for key genes and lncRNAs validation.

**Table 1:** Sequences of primers used to amplify common avian viruses.

Primer	Primer sequence (5′–3′)	Length/bp	Reference
name			
ALV-J	F: GGATGAGGTGACTAAGAAAG	545	Smith et al., 1998
	R: CGAACCAAAGGTAACACACG		
ALV-E	F: GGATGAGGTGACTAAGAAAG	197	Smith et al., 1998
	R: GGGAGGTGGCTGACTGTGT		
MDV	F: GCCTTTACACAAGAGCCGAG	507	Ding et al., 2007
	R: TTTATCGCGGTTGTGGGTCATG		
REV	F: CATACTGGAGCCAATGGTT	253	Ji et al., 2001
	R: AATGTTGTAGCGAAGTACT		

### High-Throughput Sequencing

Total RNA was extracted from three ALV-J-positive spleen samples and three ALV-J-negative samples using the TRIzol extraction method (Connolly et al., 2006).

For RNA-Seq, all RNA samples with an RIN value (Agilent 2100 score) >7.0 and a 260 nm: 280 nm optical density ratio between 1.8 and 2.2 were selected for library construction and deep sequencing. Genomic DNA and rRNA were removed, and mRNA containing poly (A) segments was enriched using oligo (dT) magnetic beads and then chemically fragmented into short fragments. The fragmented mRNA was used as a template and reverse transcribed into cDNA. Thereafter, purification, end repair, and Illumina adaptor ligation of the cDNA were performed. After selecting fragments of the desired length and amplification by PCR, the amplified cDNA libraries were qualified using an Agilent 2100 Bioanalyzer (Agilent, Santa Clara, CA, United States). These samples were subsequently sequenced on an Illumina HiSeq^TM^ 2500 instrument (Illumina, San Diego, CA, United States).

For small RNA-Seq, small RNA fractions were ligated to 5′ and 3′ RNA adaptors. Subsequently, reverse transcription and PCR were conducted to construct the cDNA library (150 bp), and then the libraries underwent quantification and quality assessment using a Qubit^®^ 2.0 fluorometer and an Agilent 2100 Bioanalyzer (Agilent). Finally, the libraries were sequenced on an Illumina HiSeq^TM^ 2500 instrument (Illumina).

### Raw Data Processing, Annotation, and Differential Expression Analysis

For RNA-Seq quality control (QC), the raw reads were subjected to FastQC analysis^1^. Clean reads were obtained by filtering out low-quality reads (*Q* scores < 20), reads with adaptor contamination, and reads with poly-*N* > 35 bp with NGS QC Toolkit (version: 2.3.3)^2^ (Hansen et al., 2010). Subsequently, the filtered reads were mapped to the chicken reference genome (*Gallus gallus* 4.0, April 2013, Ensembl Build 85) with TopHat (version: 2.0.9) (Trapnell et al., 2009) and no more than two mismatches for each read were permitted in the alignment. The transcripts were assembled based on the genome-annotation file with Cufflinks program (Trapnell et al., 2012) (v2.2.1). The fragments per kilobase of transcript per million mapped reads (FPKM) method (Trapnell et al., 2010) was used to estimate each gene expression, and the total number of reads mapped to UniGenes were calculated and normalized to the FPKM for each gene. Differentially expressed genes were identified between different groups with the R package DESeq with a *P*-value < 0.05 and fold-change ≥2. *Cis*-regulatory target genes of differentially expressed lncRNAs were predicted with RNAplex (Tafer and Hofacker, 2008) and chosen to be within 10 kb of lncRNAs. GO and KEGG functional enrichment analyses were performed with the GOseq package, and terms and pathways with corrected *P*-value of less than 0.05 were considered as being significantly enriched.

For small RNA-Seq, the clean reads were obtained by filtering out adaptor-ligated contamination and low-quality reads (*Q* scores < 20). Reads with a length <18 nt or >41 nt were trimmed, and reads with “N” and Q20 < 80% were removed using Fastx (fastx_toolkit-0.0.13.2). Then the filtered reads were mapped to chicken reference genome, miRbase with Bowtie (Langmead and Salzberg, 2012), the Rfam databases, and Repbase to analyze the aligned-reads ratio, known miRNAs, and read classifications, as well as to remove irrelevant RNAs, such as tRNAs, snRNAs, rRNAs, and cRNA. Unaligned miRNA sequences were used to predict novel miRNAs by comparing the miRNA sequences and RNAfold with those of other homologous species using miRNADeep2 program. Transcripts per million values were used to estimate the expression of each miRNA. Differentially expressed miRNAs were identified between different groups with the R package DESeq program with a *P*-value < 0.05 and a fold-change ≥2. The target genes of differentially expressed miRNAs were predicted using Miranda algorithm (Enright et al., 2003; John et al., 2004) (threshold parameters: S ≥ 150, Δ G ≤ -30 kcal/mol, and demand strict 5′ seed pairing). GO and KEGG analyses were conducted as above.

### Co-expression and ceRNA Network Construction

Co-expression analysis was performed based on the PCC between mRNAs and lncRNAs, and a co-expression network was selected using parameters PCC ≥ 0.99 or ≤-0.99, and *P* < 0.05. The lncRNAs and mRNAs with a significant positive correlation (PCC ≥ 0.99, and *P* < 0.05) were subjected to ceRNA analysis. Potential MREs were searched in lncRNA and mRNA sequences with miRanda software, and lncRNA and mRNA sequences shared the same MREs was considered to predict lncRNA–miRNA–mRNA interactions. miRNA-lncRNA/mRNA co-expression was identified when PCC ≥ 0.7 or ≤-0.7 and *P*-value < 0.01. GO and KEGG enrichment analyses were conducted with all coding genes in the ceRNA network and *P* < 0.05 was considered to reflect significantly enriched functions.

### qRT-Time PCR Analysis

qRT-PCR analysis was performed to validate the sequencing results and identify core transcripts related to ALV-J-induced tumorigenesis. qRT-PCR analysis was conducted using SYBR Premix Ex Taq^TM^ II (TaKaRa, Shiga, Japan) and an QuantStudio 5 real-time PCR instrument after cDNA synthesis with the PrimeScript^TM^ RT Reagent Kit with gDNA Eraser (TaKaRa). Primer sets were designed with Primer-BLAST^3^, and synthesized by Sangon Biotech (Shanghai, China). Three reference genes (GAPDH, SDHA, and RPL30) were detected as internal controls. All assays were run in triplicate (primer sequences are provided in **Table 2**).

**Table 2:** Sequences of primers used to amplify mRNAs and lncRNAs showing differential expression between infected and uninfected tissues.

Primer name	Type of primer	Primer sequence (5′–3′)
NFATC2	Forward	GAAGCCGATCGAAGAACCGA
	Reverse	TTGAGGCCACAGTCCAAGAC
IL9R	Forward	CCAACACATGGCTTTGGTCT
	Reverse	GAGGCAGAAGCTAGACAAGCA
OSMR	Forward	ACGTCGAACACGAAATACAAGC
	Reverse	CAGCAGCAATCCTTCACCAGT
CD34	Forward	ATGGCCGGGTACTTCCTGAT
	Reverse	TCAGCATCGGTAAATGGGCG
SRSF4	Forward	CGGGATGTGGAGCGTTTCTT
	Reverse	CTCAACGAAGCCATACCCGT
INPP4B	Forward	ACAGACCTCCAGAAGGGACA
	Reverse	ACACCTCAACCGGTTACACA
TCONS_00257530	Forward	TGAGGAGACGTTGTGCTGTC
	Reverse	ACAGTGCTCGCTGTGCTTAT
TCONS_00234495	Forward	ACTGCAAAAGATAGGGGGTGG
	Reverse	TTGACACAGGAATGCTGCAA
TCONS_00273421	Forward	GAGACAAAGCTGTCCCCGAA
	Reverse	TGCACAATACACTGAAAGCTGC
TCONS_00267624	Forward	AGGAGGATGCTCGTAGGGTT
	Reverse	ACACCAACTTCCAAGGCACA
TCONS_00263355	Forward	TCACAGGAAACGGGATGCTC
	Reverse	ACCTAGCAAAGCTGGGCATT
TCONS_00174072	Forward	AGCACGACTGACAAAGGAGG
	Reverse	TCTTGGACCTCTGGGACTGT
TCONS_00145310	Forward	CTTCGTTGCCACTCTGTTGG
	Reverse	GCAAGGTCACCACAGTTTGC
TCONS_00245752	Forward	TGGTGAGGTTGTAACGGCAA
	Reverse	TGCTCACAGCTCAGTCACAG
GAPDH	Forward	CGATCTGAACTACATGGTTTAC
	Reverse	TCTGCCCATTTGATGTTGC
RPL30	Forward	GAGTCACCTGGGTCAATAA
	Reverse	CCAACAACTGTCCTGCTTT
SDHA	Forward	CAGGGATGTAGTGTCTCGT
	Reverse	GGGAATAGGCTCCTTAGTG

### Statistics Analysis

Statistical analyses were performed using GraphPad Prism 6 software (GraphPad Software, Inc., San Diego, CA, United States). *P* < 0.05 was considered to represent a statistically significant difference.

## Results

### Diagnosis of the Chicken Population and Study Design

**FIGURE 1:**
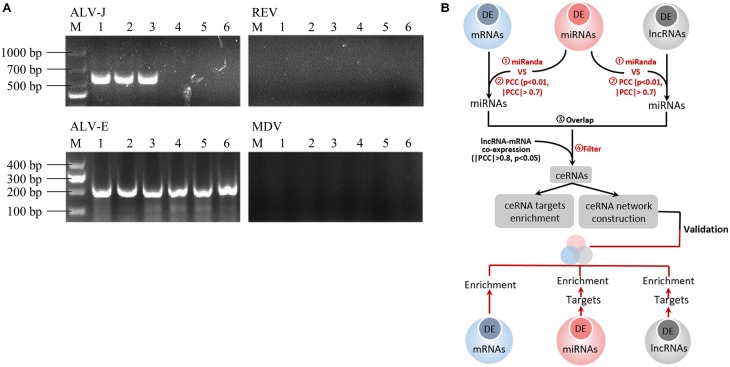
**(A)** Routine PCR and agarose gel electrophoresis for the detection of ALV-J, ALV-E, MDV, and REV. Lanes 1–3: spleen tissues from suspected ALV-J infected chickens; lanes 4–6: spleen tissues from ALV-J uninfected chickens. Lane M, DNA marker. Agarose gel electrophoresis showed a 545 bp amplicon specific to ALV-J in lines 1–3 and a 197 bp amplicon specific to ALV-J in all lines, and there is no amplicons specific to MDV and REV. **(B)** Flow-chat of the study design.

Several 20-week-old female BSFs (with or without spontaneous ALV-J infection) were reviewed, and we found three chickens with depression and hemorrhages in the phalanges skin. After dissection, we found that many tumor nodules in the liver and spleen were enlarged up to several times the normal size. Genetic diagnosis revealed three ALV-J infected chickens without MDV or REV infection, and three uninfected chickens (**Figure 1A**), which were used for sequencing analysis. A schematic representation of the study design is shown in **Figure 1B**.

### Transcriptional Landscape of mRNAs, lncRNAs, and miRNAs in ALV-J Infected Chicken

**FIGURE 2:**
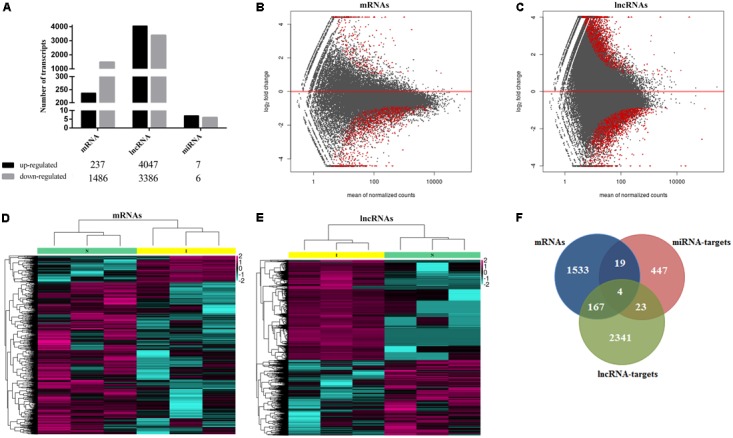
Transcriptional landscape of mRNAs, lncRNAs, and miRNAs in ALV-J infected chicken. **(A)** Number of differentially expressed mRNAs, lncRNAs and miRNAs in chicken spleens of ALV-J infected group vs. uninfected group detected by high-throughput sequence (*P*-value < 0.05 and fold-change ≥2). MA graph of mRNAs **(B)** and lncRNAs **(C)** expression levels between ALV-J infected and uninfected group. The red dots represented differentially expressed mRNAs, >0 represented upregulated and <0 represented downregulated (*P*-value < 0.05 and fold-change ≥2). Heatmaps of 1,723 significantly differentially expressed mRNAs **(D)** and 7,883 significantly differentially expressed lncRNAs **(E)** show normalized expression values. Columns represent samples, and rows represent transcripts. Colors are used to represent expression levels: above (aubergine) or below (cyan) (expression data was normalized from -2 to +2). I, ALV-J infected tissues; N, normal uninfected tissues. **(F)** Venn diagrams of differentially expressed mRNAs, miRNA targets, and lncRNA targets.

Transcriptome analysis revealed 1000s of mRNAs and lncRNAs expressed in spleen samples from ALV-J-infected chickens and uninfected chickens. We identified 9840 mRNAs in spleen samples from the uninfected chicken group and 9804 mRNAs in the ALV-J-infected group. In addition, 8266 and 9738 lncRNAs were found in uninfected and infected groups, respectively. We also identified 272 miRNAs in the uninfected group including 191 known miRNAs (∼70.2%) and 81 novel miRNAs (∼29.8%), and 259 miRNAs in the infected group including 178 known miRNAs (∼68.7%) and 81 novel miRNAs (∼31.3%) (**Table 3**). Then we compared the expression levels of transcripts (including mRNAs, lncRNAs, and miRNAs) between the ALV-J-infected group and the uninfected group, and identified 1723 mRNAs that were significantly differentially expressed in ALV-J-infected chickens, based on a fold change ≥2 and *P*-value < 0.05 (**Table 3**). Of these mRNAs, 237 (∼13.8%) were upregulated and 1,486 (∼86.2%) were downregulated (**Figure 2A**). We also identified 7,883 differentially expressed lncRNAs with 4,047 (∼51.3%) being upregulated and 3,836 (∼48.7%) being downregulated (**Figure 2A**), and only 13 miRNAs were differentially expressed in the infected group, 7 of which were up-regulated and 6 were down-regulated (**Figure 2A**).

**Table 3:** Summary of high-throughput sequencing data for spleen samples from three ALV-J-infected chickens and three uninfected chickens.

Transcripts		mRNA		lncRNA		miRNA			
Group	Samples	Total	Differentially expressed^∗^	Total	Differentially expressed^∗^	Total	Known	Novel	Differentially expressed^∗^
Uninfected Group (3)	Common	9,840		8,266		272	191	81	


	1	10,181		10,126		350	220	130	
	2	10,160		10,122		319	201	118	
	3	10,148		10,132		301	199	102	
Infected Group (3)	Common	9,804	1,723	9,738	7,883	259	178	81	13


	1	10,178		10,272		360	231	129	
	2	10,185		10,184		321	222	99	
	3	10,170		10,161		312	203	109	

^∗^*Fold change ≥2 and *P*-value < 0.05*.

MA graphs were created and scatter analyses were conducted to identify differences among mRNAs (**Figure 2B**) and lncRNAs (**Figure 2C**). We further created a heat map of differentially expressed mRNAs (**Figure 2D**) and lncRNAs (**Figure 2E**). Because there were few differentially expressed miRNAs, we did not analyze them further. Comparison of independently clustered expression profiles of lncRNAs and mRNAs revealed that both types of transcripts could be grouped into two broad classes: (I) transcripts that were present in normal uninfected tissues and decayed in ALV-J-infected tissues; and (II) transcripts that were absent or present at low levels in normal uninfected tissues and were induced at high expression levels in ALV-J-infected tissues.

By comparing differentially expressed mRNAs, miRNA targets, and lncRNA targets, we found that they shared several common genes (**Figure 2F**), including BRCA1-associated ATM activator 1 (*BRAT1*); signal peptide, CUB, and EGF-like domain 3 (*SCUBE3*); Unc-51-like kinase 3 (*ULK3*); Cathepsin B (*CTSB*); and some ATPase-related and DNA repair-associated genes. The BRAT1 protein participates in DNA damage responses, cell growth, and apoptosis by interacting with the tumor-suppressor protein BRCA1 (breast cancer 1) and the ATM protein (ataxia telangiectasia mutated) in humans (Aglipay et al., 2006; So and Ouchi, 2011). SCUBE3 was reported to modulate cancer progression by binding to TGFBR2 and activating TGFB signaling in human lung cancer cells (Wu et al., 2011). ULK3 serves as a regulator in sonic hedgehog (SHH) signaling and autophagy in humans (Young et al., 2009; Maloverjan et al., 2010). CTSB was found to participate in autophagy and immune resistance, which could be a potentially biomarker for various cancers (Bao et al., 2013; Mirkovic et al., 2015; Bian et al., 2016).

### Chicken lncRNAs Are Shorter Than Protein-Coding Genes

**FIGURE 3:**
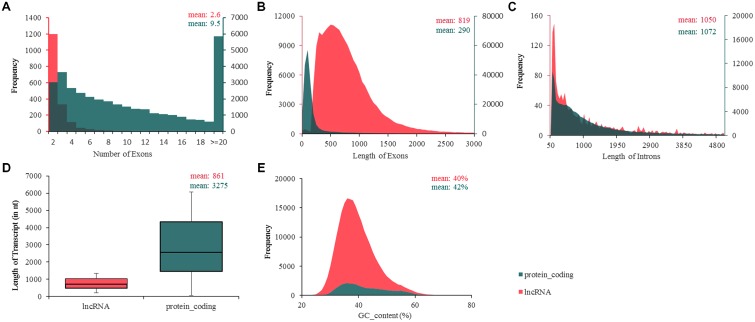
Ladscape of chicken transcript traits. **(A)** Number of exons, lncRNAs possess fewer exons per transcripts (∼2.6) than protein-coding transcripts (∼9.5). **(B)** Number of exons, mean exon length of 819 nt for lncRNAs; 290 nt for protein-coding transcripts. **(C)** Length of introns, intron length of lncRNAs on average was almost equal to protein-coding transcripts. **(D)** Transcript length, chicken lncRNAs was about one–fourth of the length of protein-coding transcripts (861 nt for lncRNAs; 3,275 nt for protein-coding transcripts). **(E)** GC content, means GC content of lncRNAs (about 40%) and protein-coding transcripts (about 42%) were almost equal. lncRNA shows in red and protein-coding transcripts in green.

More studies in mammals and zebrafish showed that lncRNAs are shorter than protein-coding transcripts and have fewer exons (Guttman et al., 2010; Cabili et al., 2011; Pauli et al., 2012; Qiu et al., 2017). In this study, to investigate whether avian lncRNAs possess similar features, we analyzed their structures, including the numbers of exons; the lengths of exons, introns, and transcripts; and the GC contents of the lncRNAs, and compared these characteristics to those of protein-coding transcripts (**Figure 3**). The results showed that, on average, chicken lncRNAs have fewer exons per transcript (∼2.6) than coding transcripts do (∼9.5) (**Figure 3A**), but a larger exon length (mean exon length of 819 nt for lncRNAs; 290 nt for protein-coding transcripts) (**Figure 3B**). However, the average intron length of lncRNAs (∼1,050 nt) was almost equal to that of protein-coding transcripts (∼1,072) (**Figure 3C**). Transcript-length analysis showed that the mean length of chicken lncRNAs was about one–fourth that of protein-coding transcripts (mean length of 861 nt for lncRNAs; 3,275 nt for protein-coding transcripts) (**Figure 3D**). In addition, the average GC content of lncRNAs (∼40%) was almost equal to that of protein-coding transcripts (∼42%) (**Figure 3E**). These findings are similar to that of zebrafish and human lncRNAs (Cabili et al., 2011; Pauli et al., 2012), and consistent with our previous study involving two ALV-J-infected chicken cell lines (Qiu et al., 2017).

### Verification of Differentially Expressed Transcripts by qRT-PCR

**FIGURE 4:**
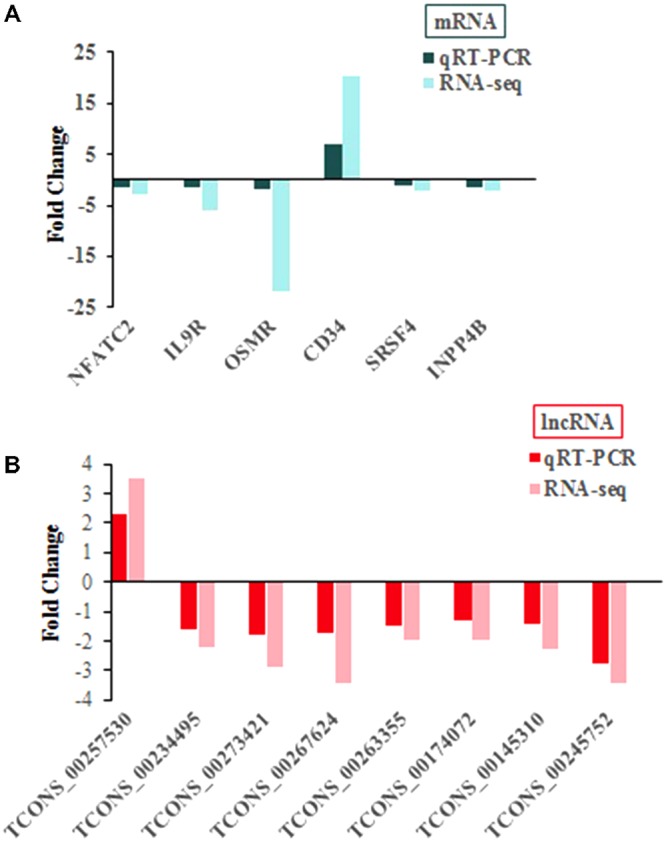
qRT-PCR confirmation of sequencing results in chickens with or without ALV-J infection. **(A)** For mRNA. **(B)** For lncRNA.

As detecting such massive differentially expressed transcripts is difficult, we selected six mRNAs (**Figure 4A**) and eight lncRNAs (**Figure 4B**) from the list of differentially expressed transcripts randomly to validate sequencing results, and uninfected group used as control group, with values <0 indicating downregulated expression and values >0 indicating upregulated expression. The results showed that both two methods showed consistent results in terms of the transcripts upregulation or downregulation (**Figure 4**).

### GO and KEGG Enrichment Analyses of the Targets of the Differentially Expressed Transcripts

**FIGURE 5:**
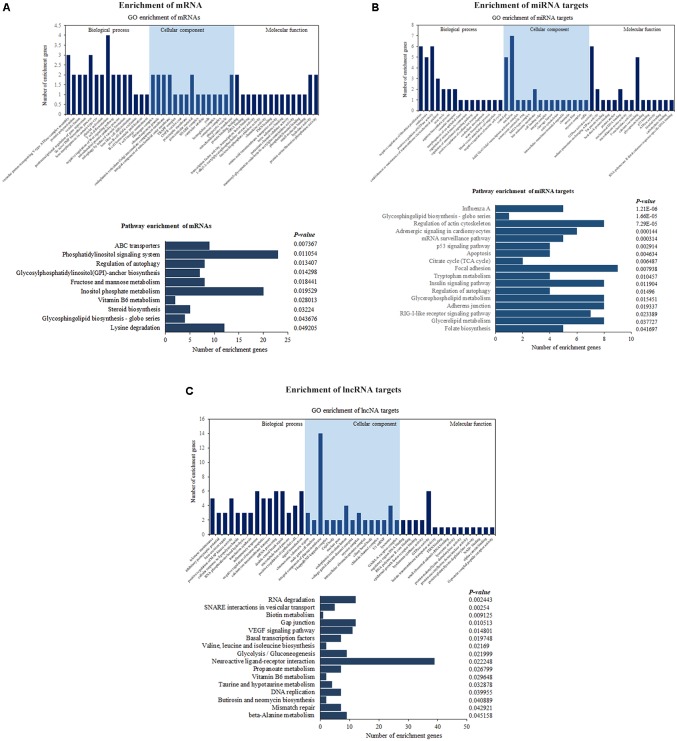
Enrichment analyses of differentially expressed mRNAs **(A)**, lncRNA targets **(B),** and miRNA targets **(C)** between ALV-J-infected and uninfected samples. Enriched GO terms, pathways and the number of genes are shown.

Functional annotation was performed by GO and KEGG pathway analyses to determine the biological significance of the whole set of differentially expressed genes, as well as two subsets of genes that were potentially targeted by differentially expressed lncRNAs/miRNAs in ALV-J-infected chickens. GO enrichment analysis of differentially expressed mRNAs genes involved in several immune-related terms, including T cell differentiation, T cell lineage commitment, B cell homeostatic proliferation, and negative regulation of Wnt signaling pathway, and KEGG pathway enrichment analysis of significantly dysregulated mRNAs showed that they were associated with 10 pathways, including regulation of autophagy, phosphatidylinositol signaling system and glycosphingolipid biosynthesis – globo series (**Figure 5A**). The details of these GO terms and pathways are provided in **Supplementary Table S1**. In addition, GO enrichment analysis revealed that miRNA targets were associated with GTPase activator activity, p53 binding, ATPase activity, and negative regulation of mitotic cell cycle. Besides, there are 17 pathways were significantly enriched, including RIG-like receptor signaling pathway, regulation of autophagy, apoptosis, p53 signaling pathway, influenza A, and glycosphingolipid biosynthesis – globo series (**Figure 5B**). The details of these GO terms and pathways are shown in **Supplementary Table S2**. Both mRNAs and miRNA targets were associated with regulation of autophagy and glycosphingolipid biosynthesis – globo series, suggesting that they may be involved in the process of ALV-J infection in chickens.

Similarly, enrichment analyses were performed for genes targeted by differentially expressed lncRNAs, which revealed positive regulation of MAP kinase activity, inflammatory response, I-kappa B/NF-kappa B complex, and other GO terms, as well as the VEGF signaling pathway, basal transcription factors, and other pathways. The VEGF signaling pathway can active multiple downstream pathways, including the Ras/MAPK pathway and the FAK/paxillin pathway, which are involved in cell proliferation, apoptosis, and survival regulation (**Figure 5C**). The details of these GO terms and pathways are shown in **Supplementary Table S3**.

### Construction of the Co-expression and ceRNA Networks

**FIGURE 6:**
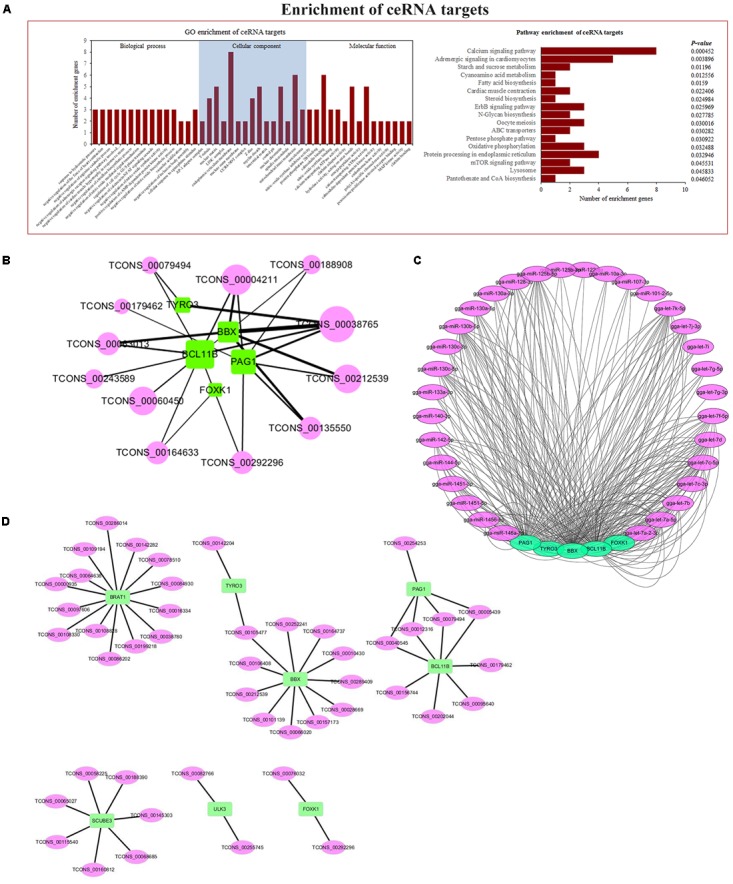
Co-expression and ceRNA network. **(A)** GO and KEGG enrichment analyses based on genes related to network. **(B)** ALV-J-induced tumor-associated ceRNA network. **(C)** ceRNA network-related miRNAs. **(D)** Co-expression network, analyzed by calculating PCC between differentially expressed lncRNAs and mRNAs.

Through ceRNA analysis, we constructed mRNA–lncRNA crosstalk networks and then performed enrichment analyses based on genes related to the networks (**Figure 6A**). We identified significant enrichments in cell-growth and cancer-associated pathways, such as the mTOR signaling pathway and the ErbB signaling pathway. The details of these GO terms and pathways are shown in **Supplementary Table S4**. Based on enrichment results and the ceRNA network, we found 267 immune-related and tumor-associated lncRNA–miRNA–mRNA co-expression links (**Figure 6B**), with multiple lncRNAs serving cooperatively as ceRNAs that got our attention which could potentially serve as biomarkers for ALV-J-induced tumorigenesis. The target genes included phosphoprotein associated with glycosphingolipid-enriched microdomains 1 (*PAG1*), HMG box transcription factor (*BBX*), forkhead box protein K1 (*Foxk1*), tyrosine-protein kinase receptor (*TYRO3*), B-cell lymphoma/leukemia 11B (*Bcl11b*), among others. PAG1 was reported to negatively regulate T-cell antigen receptor in humans (Brdicka et al., 2000). BBX is necessary for cell cycle progression from G1 to S phase (Sanchez-Diaz et al., 2001). Foxk1 was reported to be involved in cell cycle progression, to promote proliferation in myogenic progenitor cells, and to regulate Wnt/beta-catenin signaling (Shi et al., 2012). TYRO3 participates in regulating plentiful physiological processes (such as cell migration, survival, and differentiation) and in inhibiting innate immune responses mediated by Toll-like receptors by activating STAT1 (Cosemans et al., 2010). BCL11B was reported as a important regulator of in both survival and differentiation of thymocyte development in mammals, and a tumor-suppressor protein associated with T-cell lymphomas, and to play a role in the p53-signaling pathway (Wakabayashi et al., 2003). The related miRNAs are shown in **Figure 6C**, and the most of them are let-7 family, which is reported to be involved in cancer (Roush and Slack, 2008).

We constructed an mRNA–lncRNA co-expression network based on the correlation analysis between differentially expressed lncRNAs and mRNAs. We focused on 4 core genes filtered by Venn diagram analysis and 5 core genes filtered by ceRNA and enrichment analysis (**Figure 6D**).

### Validation of Core Transcripts in Tumor Tissues and Adjacent Normal Tissues

**FIGURE 7:**
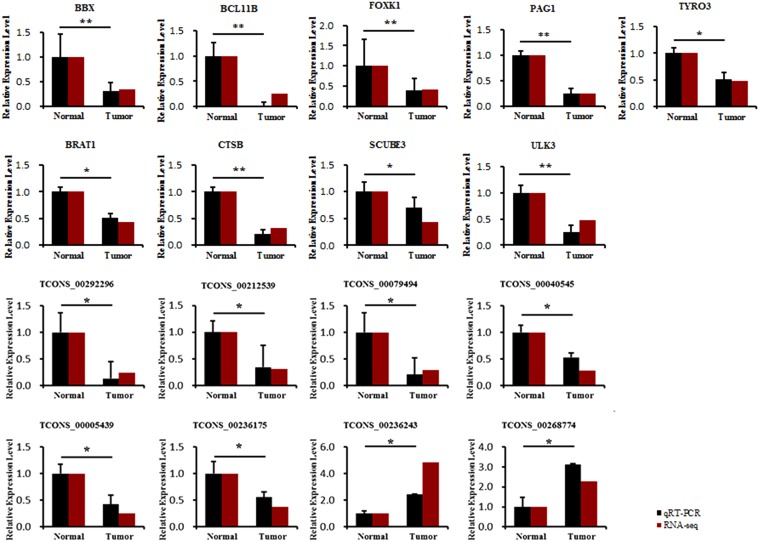
Comparison of the expression levels of nine core genes and eight related lncRNAs between liver tumor and adjacent normal tissues and then compared with RNA-seq results. ^∗^Means significant difference between tumor and adjacent normal tissues (*P* < 0.05), ^∗∗^means highly significant difference between each other (*P* < 0.01).

To research the relationship between core transcripts filtered by the above methods and ALV-J-induced tumorigenesis, we obtained independent chickens to collect liver tumors and adjacent normal tissues from four adult chickens that were diagnosed as ALV-J infection (provided by College of Veterinary Medicine, Yangzhou University, Yangzhou, China) using pathological and genetic ways. The expression of 9 genes and 8 lncRNAs was analyzed with qRT-PCR (**Figure 7**). The qRT-PCR results showed that *BBX, BCL11B, FOXK1, PAG1, TYRO3, BRAT1, CTSB, SCUBE3, and ULK3* expression levels significantly decreased after ALV-J infection, which was consistent with the RNA-Seq results. The expression levels of 8 lncRNAs (TCONS_00292296, TCONS_00212539, TCONS_00079494, TCONS_00040545, TCONS_00005439, TCONS_00236175, TCONS_00236243, and TCONS_00268774) also significantly decreased compared to those in ALV-J-infected tissues, and these results were consistent with those of RNA-Seq analysis.

## Discussion

Since ALV-J emerged, it has caused severe tumor burdens in both local breed flocks and commercial layer chickens (Gao et al., 2010). In the western world, though ALV-J has been eradicated from breeding flocks successfully, it has become more pervasive throughout China in recent decades (Payne and Nair, 2012). A recent study showed that ALV-J is an important co-infection factor in avian diseases (Dong et al., 2015).

To explore key genes, lncRNAs and ceRNA networks involved in ALV-J-induced tumorigenesis, in this study, spleen tissues of six 20-week-old female BSFs with (*n* = 3) or without (*n* = 3) spontaneous ALV-J infection were subjected to RNA-Seq and small RNA-Seq analyses. We generated a scientific annotation of the transcriptome of ALV-J-infected and uninfected chicken spleen samples. Transcriptome analysis revealed 1000s of mRNAs and lncRNAs expressed in ALV-J-infected and uninfected chicken spleen samples. The data presented here provide the first comprehensive transcriptional landscape of mRNAs, lncRNAs, and miRNAs in ALV-J-infected chickens. Through differential gene-expression analysis and Venn diagram analysis of three sets of transcripts, we found four immune or tumorigenesis-associated genes in common: including *BRAT1*, *SCUBE3*, *ULK3*, and *CTSB*. We defined a set of mRNAs and lncRNAs, characterized mRNA and lncRNA catalogs in chickens, and found that they shared many characteristics with their zebrafish and mammalian counterparts [7, 8, 25, 26]: in chickens, lncRNAs have relatively shorter transcripts and fewer exons number than mRNAs, which is consistent with our previous study (Qiu et al., 2017).

Systematic bioinformatics analysis of differentially expressed mRNAs, lncRNA targets, and miRNA targets in spleen samples from ALV-J-infected chickens and uninfected chickens revealed several highly significantly enriched GO terms and pathways. These included several immune-associated or tumor-associated terms and pathways, such as T cell differentiation, negative regulation of Wnt signaling pathway, regulation of autophagy, phosphatidylinositol signaling system and glycosphingolipid biosynthesis – globo series, GTPase activator activity, p53 binding, ATPase activity and negative regulation of mitotic cell cycle, RIG-like receptor signaling pathway, regulation of autophagy, apoptosis, p53 signaling pathway, influenza A, positive regulation of MAP kinase activity, inflammatory response, I-kappaB/NF-kappaB complex, and VEGF signaling pathway. These results were similar to previous study on ALV-J-infected HD11s (Qiu et al., 2017), which revealed pathways such as apoptosis, influenza A, and several others.

The co-expression and ceRNA network results were based on the expression of mRNAs and lncRNAs associated with ALV-J infection. Of interest, we found that the core genes *BBX, BCL11B, FOXK1, PAG1, TYRO3, BRAT1, CTSB, SCUBE3, and ULK3* may be involved in ALV-J-induced tumorigenesis. The *in vivo* expression levels showed that these core genes and their related lncRNAs (TCONS_00292296, TCONS_00212539, TCONS_00079494, TCONS_00040545, TCONS_00005439, TCONS_00236175, TCONS_00236243, and TCONS_00268774) were downregulated in liver tumors, which indicated that these lncRNAs may regulate gene expression in an enhancer-like way, as occurs with many lncRNAs in human cells (Orom et al., 2010). By validating core transcripts in tumor tissues and adjacent normal tissues, we found that *BCL11B* showed the greatest expression differences between tumor tissues and adjacent normal tissues, which attracted our attention. Many previous studies have focused on *BCL11B* and its role in carcinogenesis in humans (Bhattacharya et al., 2017); *BCL11B* ablation caused increased susceptibility to stimulation-induced carcinogenesis. Aberrant *BCL11B* expression was found to influence the proliferation, migration, and invasion of the Marek’s disease tumor cell line, MSB1 (Zhao et al., 2017), suggesting that *BCL11B* may be involved in tumorigenesis in various animals and in neoplastic diseases. Further study will be focused on validating the relationships and regulatory mechanism between *BCL11B*, its related lncRNAs, miRNAs, and the ceRNA network containing core genes, and exploring roles of this network in the ALV-J-induced tumorigenesis.

## Availability of Data and Materials

All data generated or analyzed during this study are included in this published article (and its supplementary information files).

## Author Contributions

LQ and GbC conceived and designed the experiments. ZL, LQ, and YB carried out the pre-experiment, sampling, and RNA-seq studies. LQ and XL performed the quantitative PCR studies. LQ analyzed data, drafted the manuscript, and major contributor in writing the manuscript. GhC contributed the reagents, materials, and analysis tools. All authors read and approved the final manuscript.

## Conflict of Interest Statement

The authors declare that the research was conducted in the absence of any commercial or financial relationships that could be construed as a potential conflict of interest.

## Footnotes

1 http://www.bioinformatics.babraham.ac.uk/projects/fastqc/

2 http://www.nipgr.res.in/ngsqctoolkit.html

3 http://www.ncbi.nlm.nih.gov/tools/primer-blast/

## Supplementary Material

TABLE S1 Details of these significantly enriched GO terms and pathways of differentially expressed mRNAs between ALV-J-infected and uninfected chickens.

TABLE S2 Details of these significantly enriched GO terms and pathways of differentially expressed miRNA targets between ALV-J-infected and uninfected chickens.

TABLE S3 Details of these significantly enriched GO terms and pathways of differentially expressed lncRNA targets between ALV-J-infected and uninfected chickens.

TABLE S4 Details of these significantly enriched GO terms and pathways of differentially expressed ceRNA targets between ALV-J-infected and uninfected chickens.

## Abbreviations

ALV-J: avian leukosis virus subgroup J
ceRNA: competing endogenous RNAs
FDR: false discovery rate
FPKM: fragments (a pair of reads) per kilobase of exon model per million mapped reads
GO: Gene Ontology
KEGG: Kyoto Encyclopedia of Genes and Genomes
lncRNAs: long non-coding RNAs
MDV: Marek’s disease virus
MREs: miRNA response elements
PCC: Pearson correlation coefficient
qRT-PCR: quantitative real-time polymerase chain reaction
REV: reticuloendotheliosis virus
RIN: RNA integrity number value
rRNA: ribosomal RNA
snRNA: small nuclear RNA
tRNA: transfer RNA.
